# Genome-Wide Detection of Major and Epistatic Effect QTLs for Seed Protein and Oil Content in Soybean Under Multiple Environments Using High-Density Bin Map

**DOI:** 10.3390/ijms20040979

**Published:** 2019-02-23

**Authors:** Benjamin Karikari, Shuguang Li, Javaid Akhter Bhat, Yongce Cao, Jiejie Kong, Jiayin Yang, Junyi Gai, Tuanjie Zhao

**Affiliations:** 1 Key Laboratory of Biology and Genetics and Breeding for Soybean, State Key Laboratory of Crop Genetics and Germplasm Enhancement, Soybean Research Institution, National Center for Soybean Improvement, Ministry of Agriculture, Nanjing Agricultural University, Nanjing 210095, China; benkarikari1@gmail.com (B.K.); dawn0524@126.com (S.L.); javid.akhter69@gmail.com (J.A.B.); ahszcyc@163.com (Y.C.); 2012094@gmail.com (J.K.); 2 Huaiyin Institute of Agricultural Sciences of Xuhuai Region in Jiangsu, Huai’an 223001, China; hynksyjy@163.com; 3 College of Life Science, Yan’an University, Yan’an 716000, China

**Keywords:** soybean, QTL mapping, protein content, oil content, RAD-seq, high-density bin map, main-effect QTL, epistasis

## Abstract

Seed protein and oil content are the two important traits determining the quality and value of soybean. Development of improved cultivars requires detailed understanding of the genetic basis underlying the trait of interest. However, it is prerequisite to have a high-density linkage map for precisely mapping genomic regions, and therefore the present study used high-density genetic map containing 2267 recombination bin markers distributed on 20 chromosomes and spanned 2453.79 cM with an average distance of 1.08 cM between markers using restriction-site-associated DNA sequencing (RAD-seq) approach. A recombinant inbred line (RIL) population of 104 lines derived from a cross between Linhefenqingdou and Meng 8206 cultivars was evaluated in six different environments to identify main- and epistatic-effect quantitative trait loci (QTLs)as well as their interaction with environments. A total of 44 main-effect QTLs for protein and oil content were found to be distributed on 17 chromosomes, and 15 novel QTL were identified for the first time. Out of these QTLs, four were major and stable QTLs, viz., qPro-7-1, qOil-8-3, qOil-10-2 and qOil-10-4, detected in at least two environments plus combined environment with *R*^2^ values >10%. Within the physical intervals of these four QTLs, 111 candidate genes were screened for their direct or indirect involvement in seed protein and oil biosynthesis/metabolism processes based on gene ontology and annotation information. Based on RNA sequencing (RNA-seq) data analysis, 15 of the 111 genes were highly expressed during seed development stage and root nodules that might be considered as the potential candidate genes. Seven QTLs associated with protein and oil content exhibited significant additive and additive × environment interaction effects, and environment-independent QTLs revealed higher additive effects. Moreover, three digenic epistatic QTLs pairs were identified, and no main-effect QTLs showed epistasis. In conclusion, the use of a high-density map identified closely linked flanking markers, provided better understanding of genetic architecture and candidate gene information, and revealed the scope available for improvement of soybean quality through marker assisted selection (MAS).

## 1. Introduction

The high nutritional importance of soybean is due to higher levels of protein (average 40%) and oil (average 20%) in its seed [1], which makes the cultivation of soybean central to agriculture in China and other parts of the world [2]. This crop is an important source of plant protein for human food and livestock feed as well as vegetable oil for human consumption and industrial applications [1,3]. In addition, the seed contains calcium, which benefits bone health, and isoflavones, which play a role in cancer prevention and relief of menopausal symptoms [4]. Hence, improvement of seed protein and oil content in soybean was the prime objective of soybean breeding. The phenotypic variation for protein and oil content have been reported to range 34.1–56.8% and 8.1–27.1%, respectively, in the world soybean germplasm [5], which indicates enormous potential for the improvement of soybean protein and oil contents. By comparing the modern soybean cultivars with the landraces, it is evident that traditional breeding methods have developed soybean lines with high oil content but at the cost of decreasing seed protein content or vice versa [6]. The improvement of both traits simultaneously in the same cultivar is a challenging task through conventional breeding, as protein and oil content are negatively correlated [7]. In this regard, marker-assisted selection (MAS) is a far more efficient means of achieving this by using independent or non-correlated QTLs/genes [8,9]. With this, confirmation and integration of protein and oil QTLs in soybean breeding leading cultivars with high protein and oil could increase the economic value of the crop, thereby enriching the entire value chain from farmers to processors and to the end-users [8].

Both seed protein and oil content are quantitatively inherited complex traits in soybean, and are controlled by polygenes that are very difficult to identify through conventional methods [10,11]. With the advances in molecular marker technology and statistical methods, many QTLs related to both traits have been reported over the past two decades, and there are over 240 and 322 QTLs documented for protein and oil, respectively, in the USDA Soybean Genome Database (SoyBase, http://www.soybase.org). However, only 57 of these reported QTLs have been confirmed (http://www.soybase.org). Most of these QTLs were identified by using F_2_, recombinant inbred line (RIL) and backcross inbred line (BIL) populations [3,12,13,14,15,16]. However, these genetic populations were mostly derived from two soybean parents with relatively small phenotypic differences, and hence made it difficult to effectively detect minor effects QTLs that govern significant proportion of phenotypic variance underlying both traits. Therefore, to improve the accuracy of QTL discovery, it is prerequisite to construct mapping populations by using soybean cultivars with a large phenotypic difference for the trait of interest [17]. In addition, only few QTLs, i.e., 16, linked to seed protein and oil content have been found to be stable across multiple environments and different genetic backgrounds [12,17,18]. Most of the identified QTLs for protein and oil content have been derived from North American soybean germplasm [7,12,17,18,19,20,21]. Chinese germplasm have been seldom utilized for the QTLs detection associated with protein and oil content in soybean [22]. Furthermore, mapping studies carried out earlier for both quality traits in soybean were mainly based on the identification of main-effect QTLs, and negligible efforts have been made on the study of complex genetic effects such as epistasis and environment effects [23].

The genetic architecture of complex quantitative traits is determined not only by action of genes at a single locus, but also by inter-locus and gene × environment interactions. In quantitative genetics, QTL × environment (Q × E) and epistatic interaction effects are the two major genetic components making considerable contribution to the phenotypic variation observed in complex traits [24]. The majority of the QTL mapping studies carried out used statistical methods based on single environment [25,26]. Some studies carried out in recent years have revealed Q × E effects for various traits including seed oil and protein content in soybean [22,27,28,29]. Thus, it is worth evaluating genetic attributes of soybean seed protein and oil contents in different environments. It was reported that epistatic effects often includes additive × additive variance component, hence is important even when the epistasis variance is small [30]. Jannink, et al. [31] mentioned that epistasis may also play an essential role in trait improvement even if epistatic variance components are low. The response to selection is higher and longer lasting in the presence of epistasis than its absence [32]. Therefore, QTL mapping genetic models will lead to biased estimation of QTL parameters in the case of assuming no epistasis, and therefore models that include the epistasis are proposed [31]. Many major genes are reported to exhibit inter-locus interaction in soybean [33]. In addition, the tetraploid origin of soybean makes the epistasis of great significance in this crop due to duplicate copies of genes that are likely to be interacted [34]. However, few studies have reported digenic epistatic QTL pairs for protein [15,16,22] and oil content [29]. Hence, mapping of epistatic QTLs under multiple environments is prerequisite for accurately predicting the phenotype of hypothetical-but-achievable genetic combinations.

Development of high-density genetic maps as well as their use in detection of QTLs/genes have allowed the detailed and wider understanding of the genetic basis underlying complex quantitative traits, and the analysis of genes have partitioned the related traits into individual Mendelian factors [35]. Nevertheless, there are few reports targeting mapping of QTLs related to seed protein and oil content based on the high-density map under multiple environments. Therefore, we report a high-density linkage map using RAD-seq approach, which was based on RIL population derived from two diverse soybean varieties that were tested in six different environments. By utilizing different mapping approaches: (1) main-effect and environment-specific QTLs were identified for protein and oil content; (2) related genes of major and stable QTLs were mined; and (3) analysis of epistatic QTL pairs were carried out across different environments to better elucidate the use of these QTLs for soybean seed quality improvement. The results presented here will aid marker-assisted breeding and provide detailed information for accurate QTL localization and candidate gene identification.

## 2. Results

### 2.1. Phenotypic Analysis of Seed Protein and Oil Content

**Figure 1:**
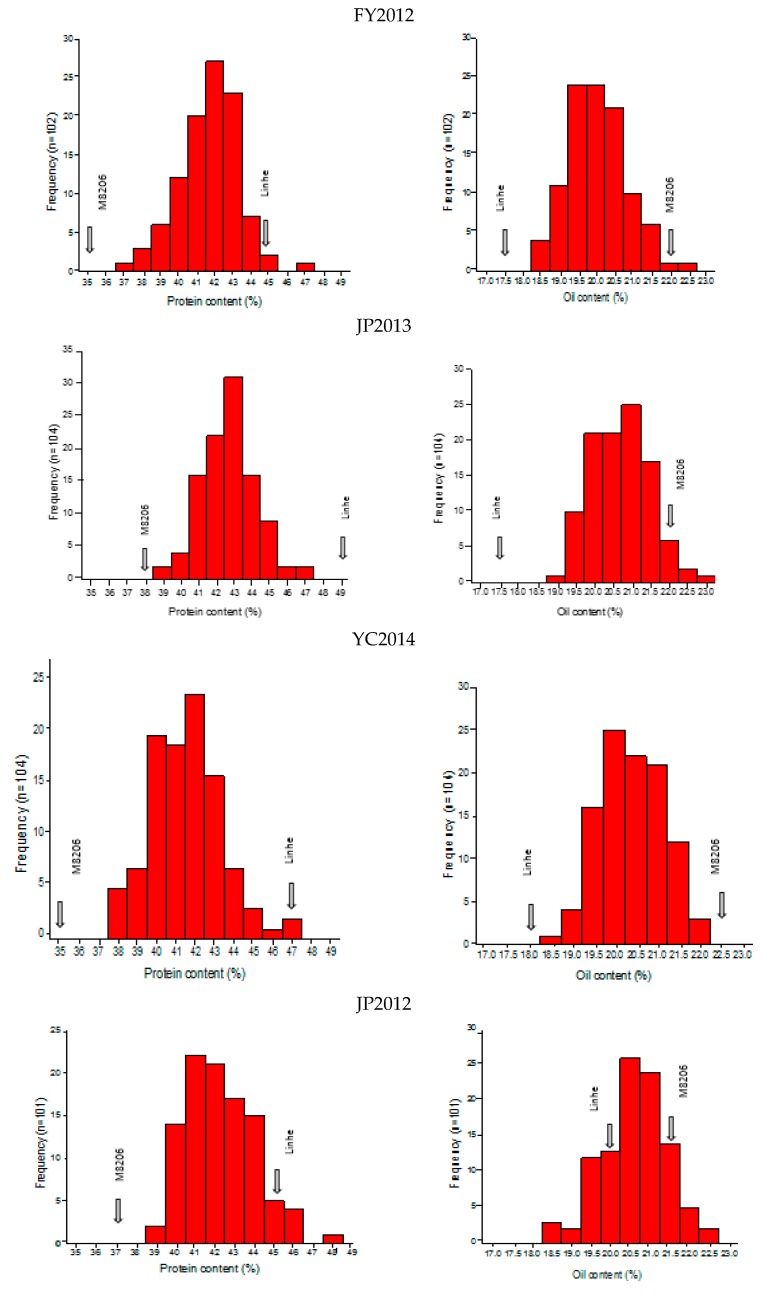

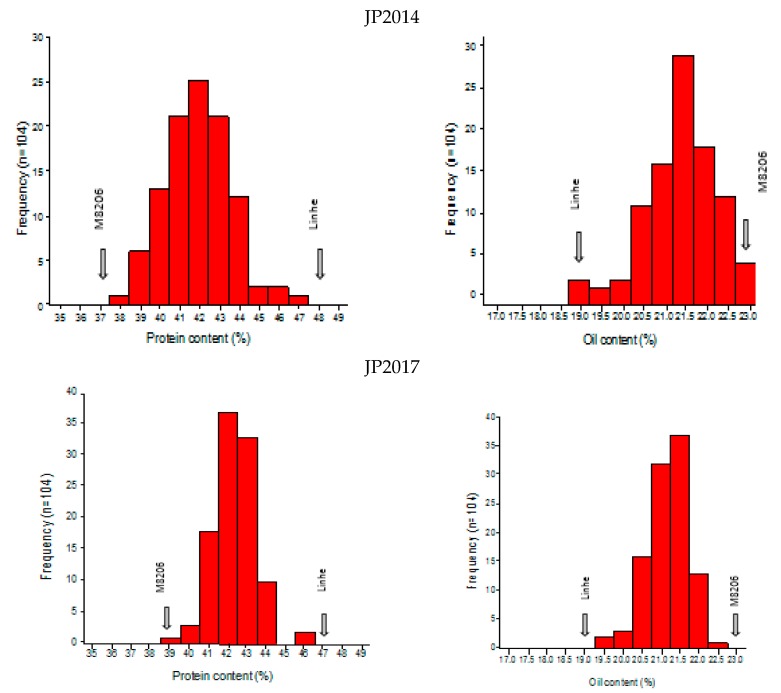
Frequency distribution of seed protein and oil content among the RILs and parents of LM6 population in six different environments (FY2012, JP2012, JP13, JP2014, YC2014 and JP2017).

Phenotypic values of protein and oil content in six environments and their multi-environment means are presented in Table S1. The phenotypic differences between the two parents for both traits were consistently high as well as substantial across all six environments and their multi-environment means (Table S1). Seed protein content of “Linhefenqingdou” was an average of about ~29.82% higher than that of “Meng 8206” across all six environments, whereas seed oil content of “Meng 8206” was about an average of ~19.82% higher than that of “Linhefenqingdou” (Figure 1 and Table S1). Several RILs exceeded their parents, Linhefenqingdou and Meng 8206, in protein and oil content respectively, which indicates that RILs showed transgressive segregation (Figure 1). In each of the six environments, kurtosis and skewness were recorded <1 and coefficient of variation (CV) <3% for both traits, which indicates that both traits are controlled by polygenes and are suitable for QTL mapping (Table S1). ANOVA results showed that the differences among RILs of mapping population were highly significant for both traits (*p* < 0.01, Table S3). The environmental differences and genotype × environment (G × E) interaction effects were also significant for both traits (*p* < 0.01, Table S2). Broad-sense heritability (H^2^) of protein content in all six environments ranged 80.20–90.60% while it varied 79.50–88.70% for oil content (Table S1). However, in the case of combined environment, the H^2^ of protein and oil content were 76.43% and 86.77%, respectively. The correlation coefficient (*r*^2^) between protein and oil content were negatively significant across all six environments and their multi-environment means (Table S1).

### 2.2. QTL Analysis for Seed Protein and Oil Content

**Figure 2:**
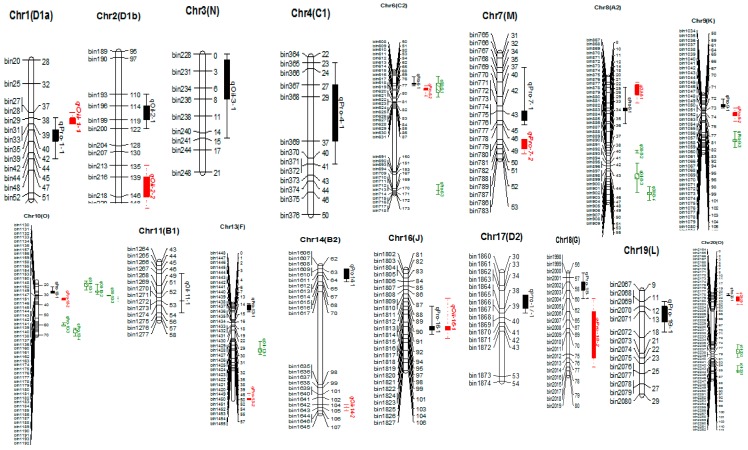
Chromosome location of the main-effect QTLs for seed protein and oil content (complete map is not presented here; this represents only the portion of the map where QTLs were identified). Right side of chromosomes indicates the interval distance between markers using cM (centiMogan) as the unit; the left side of chromosomes indicates Bin-DNA markers.

Genome-wide analyses were performed using the high-density genetic map of LM6 RIL population for the identification of QTLs related to seed protein and oil content in soybean. In total, 44 QTLs explaining 4.92–30.57% phenotypic variation (*R*^2^) associated with both protein and oil content were detected in LM6 population under all six individual environments as well as combined environment (Figure 2 and Table 1 and Table 2). For the protein content, 25 QTLs were identified on 14 chromosomes (Chr1, Chr4, Chr6, Chr7, Chr8, Chr9, Chr10, Chr13, Chr14, Chr16, Chr17, Chr18, Chr19 and Chr20) (Figure 2 and Table 1). A single QTL explained 5.74% (*qPro-14-2*) to 26.22% (*qPro-7-1*) of phenotypic variance. Among these QTLs, *qPro-7-1* were identified consistently in three environments (YC2014, JP2014 and JP2012) and combined environment, explaining an average of 19.01% of phenotypic variation. Three QTLs, viz., *qPro-7-2*, *qPro-10-1* and *qPro-10-2*, were each identified in one individual environment plus combined environment with average phenotypic variance explained (PVE) of 14.04%, 18.43% and 14.73%, respectively. In addition, one minor QTL *qPro-18-2* was also consistently identified in two environments (JP2014 and YC2014), explaining only an average of 7.75% PVE. The remaining 20 QTLs associated with protein content are environment-specific QTLs identified only in one environment (Table 1). Among all 25 QTLs identified for protein content, 10 QTLs were observed for the first time (Table 1). In total,15 QTLs were identified in the genomic region of the previously reported QTLs, of which11 were co-located in the smaller genomic regions than previously reported, which might provide more detailed information for gene identification (Table 1). Furthermore, of the 25 QTLs, 12 are major with *R*^2^ value >10%, and the other 13 are minor QTL with *R*^2^ value 8.97%. The most prominent QTL with the highest logarithm of odd (LOD) score (10.28) in individual environment was *qPro-7-1* (novel QTL) identified at a 42.01 cM position on Chr7, explaining 26.22% of phenotypic variation and displayed a positive additive effect, mainly with the positive allele from the high protein parent Linhefenqingdou. In addition, most of the QTLs showed positive additive effect with positive alleles from Linhefenqingdou, except six QTLs, viz., *qPro-4-1*, *qPro-6-3*, *qPro-13-1*, *qPro-13-2*, *qPro-19-1* and *qPro-19-2*, that displayed negative additive effect with positive alleles from low protein parent Meng 8206 (Table 1). The highest number of four QTLs for protein content were identified on Chr10, which provides information about the important role of Chr10 in governing the inheritance of seed protein content in soybean.

Nineteen QTLs were identified for seed oil content on ten chromosomes (Chr1, Chr2, Chr3, Chr6, Chr8, Chr10, Chr11, Chr13, Chr16 and Chr20) explaining 4.92–30.57% of the phenotypic variation in individual environments (Figure 2 and Table 2). Among these QTLs, the highest number of four are located on each Chr8 (*qOil-8-1*, *qOil-8-2*, *qOil-8-3* and *qOil-8-4*) and Chr10 (*qOil-10-1*, *qOil-10-2, qOil-10-3* and *qOil-10-4*), followed by three QTLs on Chr20, and the remaining seven chromosomes contain one to two QTLs each (Figure 2 and Table 2). This indicates the important roles of Chr8, Chr10 and Chr20 for regulating seed oil content in soybean. Of the 19 QTLs, 8 are major with *R*^2^ value > 10%, and the remaining 11 QTLs are minor (*R*^2^ value < 10%) (Table 3). Most prominent QTL identified in individual environments with the highest LOD score (12.11) was *qOil-10-2* (novel QTL) located at a 26.11 cM position on Chr10, explaining 30.57% of phenotypic variation, with the positive allele from high oil parent Meng 8206 (Table 2). In addition, 12 QTL showed negative additive effect with positive alleles from Meng 8206, and the remaining six QTLs displayed positive additive effect with positive alleles from low oil parent Linhefenqingdou. Among these QTLs, two QTLs on Chr10, viz., *qOil-10-2* and *qOil-10-4*, were consistently identified in three individual environments and combined environment, explaining an average of 19.85% and 19.25%, respectively, of phenotypic variation. In addition, *qOil-8-3* were consistently identified in two individual environments plus combined environment, and two QTLs, viz., *qOil-1-1* and *qOil-10-1*, were identified in two individual environments. The remaining 14 QTLs associated with oil content are environment-specific QTLs identified only in one individual environment. Among all the 19 QTLs identified for oil content, five QTLs were observed for the first time (Table 2). A total of 14 QTLs were related to the region of the QTLs reported previously, and nine of them were co-located in the regions with shorter intervals than previously reported, which would greatly assist in candidate gene identification (Table 2).

### 2.3. QTL × Environment Interaction Analysis

Seven QTLs, four for oil concentration (*qOil-8-4*, *qOil-10-2*, *qOil-11-1* and *qOil-16-1*) and three for protein concentration (*qPro-6-1*, *qPro-7-1* and *qPro-10-1*) identified on six chromosomes (Chr6, Chr7, Chr8, Chr10, Chr11 and Chr16) were found to show significant additive (*A*) and/or additive × environment interaction effects (*AE*) across different studied environments using MCIM model in QTL Network V2.1 software (Table 3). All four QTLs related to oil contributed the allele that decreased oil content through significant *A* effects, whereas all three QTLs of protein contributed an allele that increased protein content through significant *A* effects. The impact of *AE* effects of the QTLs on protein and oil content differed depending on the environments (Table 3). For example, *qOil-10-1*, an unstable QTL, could increase oil content through significant *AE* effects in JP2013 (E3) environment, but also could reduce oil content through significant *AE* effects in YC2014 (E5) environment. Similarly, the six other QTLs for seed protein and oil contents displayed similar behavior, and the instability of these QTLs was inferred to be caused by significant *AE* effects. Taken together, these seven QTLs had both significant *A* and *AE* effects (Table 3).

### 2.4. Epistatic-Effect QTLs and Epistatic QTL Interactions with the Environment

By analyzing the protein and oil content data of all six environments, three pairwise digenic epistatic QTL were identified (one for oil and two for protein) exhibiting significant epistatic effects (Table 4). One pair of epistatic QTLs for oil content are located on Chr2 and Chr13, and this QTL pair decreased oil content through significant additive × additive (*AA*) effects. Two epistatic QTL pairs for protein content, one located on Chr2 and Chr13, and another on Chr17 increased protein content through significant *AA* effects (Table 4). The epistatic effects in these QTLs could explain the proportion of phenotype variation from 0.05% to 3.81%. All three pairs of QTLs were detected to have significant additive-additive-environment (*AAE*) effects. The PVE by interaction of these epistasis QTLs with the environment (*AAE*) was from 0.03% to 0.85%. These results indicate that environment could affect the gene expression with epistatic effects on phenotype development (Table 4). However, all the main-effect QTLs were identified as not showing any epistatic effects.

### 2.5. Candidate Gene Prediction of the Major Stable QTLs

Four QTLs (*qPro-7-1*, *qOil-8-3*, *qOil-10-2* and *qOil-10-4*) of the total 44 QTLs identified for seed protein and oil contents in the present study were considered as major and stable QTLs being consistently identified in at least two environments plus combined environment as well as having R^2^ value >10%. Hence, these QTLs were of major focus, and therefore all the model genes within the physical intervals of these QTLs as well as their gene annotations were downloaded from the SoyBase (http://www.soybase.org) and Phytozome database (https://phytozome.jgi.doe.gov). In total, 192, 311, 112 and 242 model genes were present in the physical location of *qPro-7-1*, *qOil-8-3*, *qOil-10-2* and *qOil-10-4*, respectively (Table S3). Of these genes, 111 showed a relationship with protein and oil storage and/or amino acid and lipid biosynthesis and metabolism based on the gene ontology (GO) and annotation information, and they comprised 20 out of the 192 genes in *qPro-7-1*, 30 of the 311 genes in *qOil-8-3*, 24 of the 112 genes in *qOil-10-2* and 37 of the 242 genes in *qOil-10-4* (Table S4). All these candidate genes within each of these four major QTLs were screened based on their related function to protein or oil, irrespective of whether QTLs were associated to oil or protein content because these two traits are significantly negatively correlated, and it was reported that seed energy balance (Eseed = Ep + Eo + Ec, where E is energy, p is protein, o is oil, and c is carbohydrate), which is the basis for the negative correlation, hence increase or decrease in oil content may be regulated by the decrease or increase in proteins, respectively [3,48].

RNA-Seq expression data of predicated candidate genes were extracted from SoyBase (www.soybase.org) according to Severin, et al. [49], and some of these candidate genes showed high fold-change in gene expression in different soybean tissues as well as growth stages (Figure S1 and Table S6). Based on the RNA-seq analysis, Glyma07g08950 screened from *qPro-7-1* showed the highest fold change in gene expression during the seed development stage, followed by Glyma07g09790, Glyma07g09060 and Glyma07g09230, which also showed significant high gene expression in flower, pod and seed development stages, and root nodules of soybean. Out of 30 candidate genes screened from *qOil-8-3*, Glyma08g18110, Glyma08g17760, Glyma08g17610 and Glyma08g17600 were highly expressed in seed development, nodule and other reproductive tissues. In the case of *qOil-10-2*, all the genes showed low expression among the tissues during different growth stages with only Glyma10g06810 being highly expressed in the root nodules (Figure S1 and Table S5). Among the candidate genes screened for *qOil-10-4*, Glyma10g28370, Glyma10g28180, Glyma10g27980, Glyma10g26380, Glyma10g24620 and Glyma10g24590 were relatively highly expressed in the seed development stage as well as root nodules. Accumulation of protein and oil in soybean takes place mainly in seed development stage, and in addition root nodules are involved in biological nitrogen fixation (BNF) in soybean, which is a major element needed for the protein and oil formation [50,51]. Hence, these highly expressed genes can be considered as potential candidates for seed protein and oil content, which however needs further functional validation.

## 3. Discussion

Seed oil and protein content are the two economically important traits determining the quality and value of soybean. Hence, achieving soybean lines with higher protein and oil content was a primary goal of soybean breeding programs. However, to develop the improved soybean cultivars, it is imperative to have a detailed understanding of the genetic mechanism as well as genetic elements associated with trait of interest. In this regard, the present study used the high-density genetic map of RIL population derived from two diverse cultivated Chinese soybean genotypes showing large phenotypic variation for both oil and protein content, to identify the main-effect and epistatic-effect QTLs as well as their interaction with the environment. Here, parent lines “Linhefenqingdou” and “Meng 8206” exhibited consistent and large phenotypic difference for both protein (~29.82%) and oil (~19.82%) content across all six environments. Compared with other studies, the large genetic variation generated from “Linhefenqingdou” × “Meng 8206” cross in this study allowed the detection of considerable number of protein and oil QTLs with both large and small genetic effects [15,21,22,52]. Frequency distribution of both traits showed the characteristics of continuous variation (Figure 1). In this study, transgressive segregants for protein and oil content were observed in both directions, indicating that both parents contributed alleles for these traits in the RILs (Figure 1). This is in agreement with the findings of Patil et al. [7], who also reported transgressive segregants for seed protein and oil content among RILs of soybean in multiple environments. A significant variation found among the RILs for both the traits also indicated the presence of genetic diversity in the selected parents for these traits (*p* < 0.01; Table S3). Moreover, significant environmental differences and G × E interaction effects indicated that both traits are not only determined by genetic factors but also by environment and their interaction (G × E). Previous studies indicated that the estimates of heritability for oil and protein contents varied 70.0–89.0% and 56.0–92.0%, respectively, depending on the populations and environments [7,12,13,21]. In our study, the estimated heritability varied 80.0–91.0% and 79.0–89.0% for protein and oil content, respectively, in the RIL population across six different environments (Table S2), which was consistent with most previous studies. The high heritability suggests that if the trial were repeated in same growing/environment conditions there would bea high possibility of achieving the same kind of phenotypic results. The highly significant negative correlation between seed protein and oil content in soybean was in accordance with that of earlier findings [8,53,54].

Linkage mapping has been routinely used for the QTL/gene detection in crop plants, and is an efficient approach to analyze quantitative traits. The quality of genetic maps has a great influence on the accuracy of QTL detection [55,56]. In this context, high-density genetic map aided in the identification of more recombination events in a population as well as increased QTL mapping accuracy [57]. In soybean, many genetic linkage maps have been published based on restriction fragment length polymorphism (RFLP) markers, isozyme, morphological, and biochemical markers, simple sequence repeat (SSR) markers and integrated genetic map of different molecular markers Zhaoming, Xiaoying, Huidong, Dawei, Xue, Hongwei, Zhengong, Zhanguo, Jinzhu and Rongsheng [29]. With the advances in genome sequencing technology, few high-density genetic maps based on high-throughput SNP markers have been constructed for soybean [8,15,58,59,60]. In this study, we used high-density genetic map of the LM6 population that contains 2267 bin markers integrated to all 20 linkage groups (LGs), and the average distance between adjacent markers was only 1.01 cM for LM6 population. Use of high-density binmap assisted in QTL identification with tightly linked markers, and provided a good foundation for analyzing quantitative traits. Furthermore, to reduce environmental errors, RILs were planted in six environments (consisting of different locations and years), and each of the environments was statistically different. As described by Jansen, et al. [61],the QTL position and effects can be accurately evaluated if the phenotypic data are collected in various environments that are different from a statistical perspective.

Markers associated with the QTLs underlying seed protein and oil content in soybean were mapped onto all linkage groups. In total, 25 and 19 main-effect QTLs were identified for protein and oil content, respectively, using a high-density bin map based of RIL population derived from “Linhefenqingdou” × “Meng 8206” cross, and they contributed significantly to the seed protein and oil content. The QTL results of our study revealed better matches with SoyBase database (www.soybase.org; Table 2 and Table 3); however, new main-effect loci were also detected (Table 1 and Table 2). There were ten and five novel main-effect QTLs identified for protein and oil, respectively, indicating the distinct genetic architecture in the population derived from two diverse Chinese cultivated soybean genotypes. Among the ten novel protein QTLs, *qPro-7-1* was identified as a major and stable QTL related to protein content. More remarkably, these ten novel QTLs related to protein together explained more than 80% of the PV, which suggested that these loci might be potential loci for protein. It was notable that *qOil-10-2* explained the highest PV (19.85%) followed by *qOil-8-3* among the five novel QTLs identified for oil content, and both were reported as stable and major QTLs for oil content. The five novel QTLs identified for oil together explained 66.61% of the PV, which suggested potential importance of these loci for seed oil content. Hence, identification of many novel QTLs in the present study suggests the need to use more germplasm for revealing the complex genetic basis of soybean. The positive alleles of five main-effect QTLs related to protein were from the low seed protein parent Meng 8206. Similarly, positive alleles of seven main-effect QTLs related to oil were from the low seed oil parent Linhefenqingdou. Finally, it is important to note that not only the higher phenotype parent contributes positive alleles, but also the contribution of positive alleles by lower phenotype parent cannot be disregarded; similar results are also discussed in [62,63,64].

The stability of QTL is essential for the use in a breeding program. In addition to novel stable QTLs identified for both seed quality traits, 15 and 14 QTLs for protein and oil content have been previously colocalized in the same physical interval by earlier studies (see references in Table 2 and Table 4). Out of 15 colocalized protein QTLs, four major QTLs associated with protein content, viz., *qPro-9-2*, *qPro-10-1*, *qPro-13-2* and *qPro-18-1*, explained 11.94%, 18.43%, 10.78% and 10.09% of the PV, respectively (Table 2). *qPro-9-2* is reported as being associated with nearest markers ofSat_293 and BARC-010523-00698 covering large physical interval of 2,967,367–46,053,138 [14]. *qPro-10-1* has been detected as linked to the nearest marker Satt173 in the similar physical distance [14,65]. *qPro-13-2* and *qPro-18-1* were mapped in the same region as previous studies [39,40,65]. Of the 14 QTLs of oil previously reported, five are major QTLs with *R*^2^ value >10% (see references in Table 1). Hence, these QTLs might also be considered as major and stable QTLs for further fine mapping and map-based cloning to unravel the mechanisms of seed protein and oil content in soybean, as well as might be good for marker-assisted breeding.

Several QTLs of various traits can map to the same locus [47]. In this study, two pairs of QTLs, *qPro-10-1* and *qOil-10-2* as well as *qPro-16-1* and *qOil-16-1*, with inverse additive effect for protein and oil were located in the same marker interval, which implies that *qPro-10-1* and *qPro-16-1* not only control protein content in seeds but also affect oil content in seeds (Table 1 and Table 2). It supports the negative correlation between protein and oil concentration in soybean seeds [58,66].

In addition to main-effect genes, the genetic architecture of a complex trait is also regulated by inter-locus interactions as well as their interaction with the environment [67]. Understanding the additive and epistatic, QTL × environment effects of QTL and their PVE will be valuable for effective MAS, because it will greatly guide the breeder in the QTL selection and prediction of the final outcomes of MAS [31]. Previous studies reveal that seed protein and oil content in soybean is significantly affected by environment, even in early developmental stages [13,29,68]. QTLs with greater additive effects are often more stable in multiple environments and different seed developmental stages [3,7,13,68]. For example, *qPro-7-1* (additive effect: 0.59) could be identified in three environments plus combined environment; however, *qOil-16-1* (additive effect: 0.14) was found in only one environments in this study (Table 3). The genetic architecture of protein and oil content also includes epistatic interactions between QTLs [13,15]. Hence, ignoring inter-genic interaction will lead to overestimation of individual QTL effects and underestimation of genetic variance [69]. This in turn could result substantial drop in the genetic response to MAS, particularly at late generations [45]. In this study, three pairs of digeneic epistatic QTLs pairs, one for oil content and two for protein content, were identified. However, these epistatic QTLs did not display additive effects alone. They might serve as modifying genes that themselves have no significant effects but regulate the expression of protein and oil related genes through epistatic interactions. All three pairs have both significant *AA* and *AAE* effects; however, the total PVE explained by two epistatic pairs of protein was about 1.5%, whereas PVE by oil epistatic QTL pairs was 3.81%. Similar results for epistatic interaction of protein and oil QTLs have been also reported by earlier studies [13,14,16,22]. The presence of epistatic interactions for a given trait makes selection difficult. Interestingly, all main-effect QTLs identified in the present study had no epistatic interaction, which increases the heritability of the trait and makes selection easier.

In the present study, mining of the candidate genes for seed protein and oil content in soybean revealed 857model genes within the physical intervals of four major and stable QTLs. Based on the gene ontology (GO) and annotation information, a total of 111 putative candidate genes (20 in *qPro-7-1*, 30 in *qOil-8-3*, 24 in *qOil-10-2* and 37 in *qOil-10-4*) known to function, directly or indirectly, in protein and oil storage and/or amino acid and lipid biosynthesis and metabolism (Tables S3 and S4) were found. From the available gene expression data (RNA-seq), 15 of the 111 predicted candidate genes revealed significantly higher gene expression especially in the seed development stage and root nodules (Figure S1 and Table S5). It has been reported that protein and oil accumulation in soybean seed occurs particularly during seed development stage [50,70], hence genes expressed sustainably in seed development stage might affect the biological process associated with oil and protein. Secondly, high-protein soybean seed production requires a large amount of Nitrogen (N), which in most cases is largely derived from N_2_ fixation through root nodules [71]. Vollmann, et al. [72] revealed that seed protein content was drastically reduced in seasons of insufficient nitrogen fixation. Reduced BNF is also reported to decrease seed composition traits especially seed oil and protein content in soybean [51]. Therefore, the above 15 highly expressed genes identified during seed development stage and root nodules might be considered as the potential candidate genes responsible for seed protein and oil content in soybean. However, it requires further validation and verification to confirm their actual role in seed protein and oil content in soybean, as well as their use for the improvement of seed quality traits. Some of these genes were included in a future project for functional validation to ascertain their effect on the seed quality traits. Hence, the precise identification of QTLs in a specific physical interval through the use of high-density map in the present study would make it easy to find candidate genes.

## 4. Materials and Methods

### 4.1. Plant Material and Experimental Conditions

The mapping population of 104 F_7:8_–F_7:11_ RILs was advanced by single-seed descent method from the cross between Linhefenqingdou (♀) × Meng 8206 (♂) (designated as LM6). Linhefenqingdou contains high seed protein and low seed oil content, whereas Meng 8206 contains high seed oil but low seed protein. Parental accessions and RIL population were planted in a randomized complete block design (RCBD) with three replications in six environments: Fengyang Experimental Station, Chuzhou, Anhui Province (Latitude 32°87′ N; Longitude 117°56′ E), in 2012 (FY2012); Jiangpu Experimental Station, Nanjing, Jiangsu Province (Latitude 33°03′ N; Longitude 118°63′ E), in 2012, 2013, 2014 and 2017 (JP2012, JP2013, JP2014 and JP2017); and Yancheng Experimental Station, Yangcheng, Jiangsu Province (Latitude 33°41′ N; Longitude 120°20′ E), in 2014 (YC2014). Standard cultural and agronomic practices were used for the cultivation of soybean crop in each environment [66,73].

### 4.2. Measurement and Analysis of Seed Protein and Oil Contents

For the estimation of protein and oil contents in soybean seed, 18–20 gram sample of seed were analyzed with an Infratec^TM^1241 near infrared analysis (NIR) Grain Analyzer (Foss, Hillerød, Denmark) following Li et al. [6]. The protein and oil values were converted to a moisture-free basis. Phenotypic data of seed protein and oil contents were estimated for each RIL as well as their parents in three replications for all six environments.

Descriptive statistics such as mean, standard deviation (SD), maximum and minimum trait value, coefficient of variation (CV%), skewness and kurtosis, as well as analysis of variance (ANOVA) and heritability among RILs and parents for seed protein and oil content and correlations among pairs of traits, were calculated using the SPSS17.0 software (http://www.spss.com) according to Palanga et al. [74]. Frequency distribution of phenotypic data for each environment was plotted using Origin 9.0 Statistical Software (Origin Corporation, Northampton, MA, USA).

### 4.3. Bin Map Construction

Genomic DNA for the map construction were extracted from the young leaves of LM6 mapping population and their parents following the protocol of Zhang et al. [75]. *Taq* Ienzyme was used to digest this genomic DNA for constructing genomic DNA library following Baird, et al. [76]. DNA fragments of 400–700 bp were selected and sequenced using the Illumina HiSeq 2000 standard protocol for multiplexed shotgun genotyping (MSG), and 90-mer paired-end reads were generated [77]. The sequenced reads were aligned to the Williams 82 reference genome using the SOAP2 software [78]. Single Nucleotide Polymorphism (SNP) calling and genotyping were conducted using Real SFS software [79], based on the Bayesian estimation. Subsequently, using a three-standard filter, 50 < depth < 2500, a probability of site mutation 95%, and every SNP loci separated by at least 5 bp, we obtained high confidence SNPs.

Bin markers are a type of genomic markers, that have been derived from SNP markers. A slightly modified sliding-window approach proposed by Huang et al. [80] was used to construct bin markers based on the SNP dataset without imputation. A window size of 15 SNPs and a step size of 1 SNP were used to scan consecutive SNPs. Windows with 11 or more SNPs from either parent were considered homozygous but those with fewer SNPs from a single parent were considered heterozygous. SNP positions that switched from one genotype to another consecutive genotype were used to determine recombination breakpoints. Consecutive intervals of 30-kb that did not possess any recombination event within the population were combined into bins, and these bins were used as markers. According to the breakpoint information, the bin information was generated using a PERL script [81]. The linkage maps of bin markers were constructed for the RIL population using JoinMap 4.0 [82]. The high-density genetic map of the LM6 population contained 2267 bin markers. The total length of this map was 2453.789 cM and the average distance between the markers was 1.08 cM (Table S6). The average length of each linkage group was 122.67 cM, and the mean marker number of each linkage group was 113 (Table S6).

### 4.4. Mapping of Main- and Epistatic-Effect *QTLs*

Main-effect QTLs were detected using WinQTL Cart 2.5 software with the model of composite interval mapping (CIM) [83]. The window size, working speed, control marker number and permutation times were set at 10 cM, 1 cM, 5 cM and 1000 cM, respectively, in CIM model for all six environments. Treatment differences were determined at α of 0.05. CIM model was also used to identify the main-effect QTLs for the combined environments by using the same above parameters as set in the individual environments. QTLs detected in different environments at the same, adjacent, or overlapping marker intervals were considered the same QTL [29,84,85]. Location of main-effect QTLs on each chromosome/linkage group was drawn by using MapChart 2.1 software [86].

QTL genetic-effects including additive, additive × additive epistatic-effects and their environmental interaction effects in the RIL population were analyzed according to the method of Wang, et al. [87]. The mixed-model based composite interval mapping (MCIM) model in QTL Network V2.1 software [88] was used for the estimation of these effects; in addition, the critical *F*-value of the MCIM model was calculated with 10,000 permutation tests. QTL effects were estimated using the Markov Chain Monte Carlo (MCMC) method with 20,000 Gibbs sampler iterations and candidate interval selection and putative QTL detection, and the QTL effects were calculated with an experiment-wise type I error under α = 0.001 [35,89,90]. In this study, we analyzed the protein and oil contents from all six environments plus the combined environment.

### 4.5. Identification of Candidate Genes

QTL identified in at least two environments plus combined environment with *R*^2^ value >10% were considered as major and stable QTLs [29] Soybean genomic data were downloaded from the Phytozome (http://phytozome.jgi.doe.gov) and SoyBase (http://www.soybase.org) website, according to the physical interval position of the major and stable QTLs, and candidate genes were extracted from the predicted gene list based on the gene annotations (http://www.soybase.org; https://phytozome.jgi.doe.gov) as well as previously published literature. The predicted candidate genes were further screened using the gene ontology (GO) information obtained from SoyBase through online resources: GeneMania (http://genemania.org/), Gramene (http://archive.gramene.org/db/ontology), Kyoto Encyclopedia of Genes and Genomes website (KEGG, www.kegg.jp) and National Center for Biotechnology Information (NCBI: https://www.ncbi.nlm.nih.gov). RNA-Seq dataset available at SoyBase website was used to analyze the expression of predicted candidate genes in different soybean tissues as well as development stages. A heat map for fold-change in expression of these predicted candidate genes was constructed using R Package (http://www.R-project.org/).

## 5. Conclusions

In this study, we used high-density genetic map of LM6 RIL population (Linhefenqingdou × Meng 8206) to identify QTLs associated with seed protein and oil content in soybean. A total of 44 main-effect QTLs related to both traits were identified, and four of them were major and stable QTLs identified in at least two environments plus combined environments. In addition, of these 44 QTLs, 15 QTLs were novel reported for the first time, 29 QTLs were coincident with previous research and most of them have narrowed physical distance. Based on RNA-seq analysis, 15 genes within the physical interval of four major and stable QTLs involved directly or indirectly in seed protein and oil biosynthesis/metabolism processes were highly expressed during seed development stage and root nodules that might be considered as the potential candidate genes. Furthermore, seven QTLs showed significant Q × E interaction effects, and three digenic epistatic QTLs pairs were identified. However, no main-effect QTLs showed epistasis, which increases the heritability of the trait and makes selection easier. Our findings might be of great usefulness for marker-assisted breeding, and could provide detailed information for accurate QTL localization and candidate gene discovery.

## Figures and Tables

**Table 1:** Main-effect QTLs identified for seed protein content in the LM6 RIL population across the six environments and combined environment.

QTLs Names ^a^	Chr ^b^	Pos (cM) ^c^	LOD ^d^	*R*^2^ (%) ^e^	A ^f^	Confidence Interval (cM) ^g^	Env. ^h^	Ref. ^i^
*qPro-1-I*	1	39.51	2.74	6.32	0.32	37.9–44.4	CE	[18]
*qPro-4-1*	4	37.51	2.55	5.80	−0.45	27.1–41.4	YC2014	New
*qPro-6-1*	6	62.11	6.17	15.09	0.74	56.1–65.4	YC2014	New
*qPro-6-2*	6	67.41	5.08	13.23	0.69	65.4–74.8	YC2014	New
*qPro-6-3*	6	168.61	3.47	11.16	−2.58	163.5–172.7	JP2012	New
*qPro-7-1*	7	41.71	5.62	13.59	0.69	34.2–44.7	YC2014	New
		42.01	10.28	26.22	0.81	40.9–42.6	JP2014	
		42.01	8.46	22.21	0.58	38.8–43.4	CE	
		44.91	4.34	14.04	2.85	42.9–46.1	JP2012	
*qPro-7-2*	7	49.21	5.30	15.01	0.64	48.8–51.9	JP2014	[7]
		49.21	4.59	13.07	0.45	48.8–52.0	CE	
*qPro-8-1*	8	19.91	2.60	8.21	2.15	12.4–25.9	JP2012	New
*qPro-9-1*	9	67.41	3.51	7.70	0.46	61.6–69.5	YC2014	[14]
*qPro-9-2*	9	74.31	3.54	11.94	0.39	70.3–81.8	JP2017	[14]
*qPro-9-3*	9	97.71	2.78	6.40	0.33	91.3–105.4	CE	[14]
*qPro-10-1*	10	26.11	8.93	21.52	0.76	22.3–27.5	YC2014	[36]
		26.11	6.26	15.35	0.49	23.1–28.4	CE	
*qPro-10-2*	10	33.31	5.46	13.62	0.48	32.9–33.9	CE	New
		34.01	6.18	15.84	0.66	33.1–35.3	YC2014	
*qPro-10-3*	10	59.51	3.66	8.10	0.46	58.0–61.3	JP2014	[37]
*qPro-10-4*	10	64.71	3.53	7.84	0.45	62.7–70.8	JP2014	[37]
*qPro-13-1*	13	0.91	3.24	10.27	−2.11	0.0–06.6	JP2013	[38]
*qPro-13-2*	13	79.91	3.05	10.78	−2.50	75.6–81.1	JP2013	[39]
*qPro-14-1*	14	63.41	2.70	8.46	0.32	62.9–67.0	JP2017	[40,41]
*qPro-14-2*	14	104.51	2.66	5.74	0.38	104.4–105.5	JP2014	[42]
*qPro-16-1*	16	94.71	3.20	6.96	0.42	89.2–97.2	JP2014	New
*qPro-17-1*	17	38.21	3.99	9.32	0.57	34.7–39.3	YC2014	New
*qPro-18-1*	18	57.51	4.52	10.09	0.50	56.4–61.6	JP2014	[40]
*qPro-18-2*	18	64.91	3.11	7.15	0.42	62.2–69.2	JP2014	[14]
		73.51	3.57	8.33	0.47	67.5–77.9	YC2014	
*qPro-19-1*	19	11.91	3.52	8.05	−0.55	10.7–17.2	YC2014	[39]
*qPro-20-1*	20	2.01	3.34	10.71	2.49	0.0–2.9	JP2012	New

^a^ QTLs detected in different environments at the same, adjacent, or overlapping marker intervals were considered the same QTL; ^b^ Chromosome; ^c^ Position of the QTL; ^d^ The log of odds (LOD) value at the peak likelihood of the QTL; ^e^ Phenotypic variance (%) explained by the QTL; ^f^ Indicates additive, those with positive values show beneficial alleles from parent Linhefenqingdou while those with negative values show beneficial alleles from parent Meng 8206; ^g^ 1-LOD support confidence intervals (confidence interval length); ^h^ Environment where CE represents combined environments and others refer materials and methods; ^i^ References from www.soybase.org.

**Table 2:** Main-effect QTLs for seed oil content in the LM6 RIL population across the six environments and combined environment.

QTLs Names ^a^	Chr ^b^	Pos (cM) ^c^	LOD ^d^	*R*^2^ (%) ^e^	A ^f^	Confidence Interval (cM) ^g^	Env. ^h^	Ref. ^i^
*qOil-1-1*	1	39.31	4.88	10.58	−0.29	37.4–39.5	JP2014	[33]
		40.01	4.13	10.14	−0.24	37.9–43.3	YC2014	
*qOil-2-1*	2	139.21	4.08	13.31	1.38	138.0–149.4	JP2012	[43]
*qOil-2-2*	2	114.01	3.10	7.73	0.23	110.8–121.9	JP2013	[14]
		97.61	2.55	4.92	0.13	94.7–110.9	CE	
*qOil-3-1*	3	6.11	2.83	8.96	1.09	0.9–15.4	JP2012	[14]
*qOil-6-1*	6	62.11	2.74	5.44	−0.71	55.0–75.6	FY2012	New
*qOil-8-1*	8	11.41	2.77	6.03	0.14	09.7–17.6	CE	[43]
*qOil-8-2*	8	36.71	2.93	7.98	−0.16	36.3–37.0	CE	New
*qOil-8-3*	8	42.81	4.08	10.49	−0.24	40.7–43.8	YC2014	New
		45.51	2.88	6.19	−0.21	43.8–51.7	JP2014	
		46.91	7.91	19.37	−0.25	44.2–49.4	CE	
*qOil-8-4*	8	51.81	4.70	12.69	−0.27	50.1–55.2	YC2014	[44]
*qOil-10-1*	10	17.61	3.54	9.64	−0.23	16.1–19.3	YC2014	[14]
		19.31	2.57	7.48	−0.15	17.3–24.8	JP2017	
*qOil-10-2*	10	23.01	3.70	10.94	−0.27	19.0–26.1	JP2013	New
		26.11	12.11	30.57	−0.48	25.4–27.9	JP2014	
		26.11	6.62	16.91	−0.30	20.6–28.6	YC2014	
		26.11	8.41	21.00	−0.26	22.9–28.6	CE	
*qOil-10-3*	10	30.41	3.70	9.90	−0.29	30.4–30.8	JP2013	[14,45]
*qOil-10-4*	10	32.91	5.66	14.50	−0.32	32.2–33.6	JP2013	[14]
		33.31	6.06	15.65	−0.29	32.9–35.3	YC2014	
		33.31	7.66	19.42	−0.25	32.9–35.6	CE	
		33.91	10.53	27.49	−0.45	33.2–34.7	JP2014	
*qOil-11-1*	11	52.91	4.85	12.61	−0.31	46.0–55.5	JP2013	[43]
*qOil-13-1*	13	38.31	3.35	10.01	0.19	32.3–43.1	JP2017	[46]
*qOil-16-1*	16	94.71	3.92	8.69	−0.17	87.7–97.8	CE	New
*qOil-20-1*	20	4.41	3.09	9.86	1.20	0.0–13.8	JP2012	[47]
*qOil-20-2*	20	72.41	3.15	9.27	0.17	66.3–81.8	JP2017	[14]
*qOil-20-3*	20	99.21	3.92	8.28	−0.25	92.7–102.2	JP2014	[14]

^a^ QTLs detected in different environments at the same, adjacent, or overlapping marker intervals were considered the same QTL; ^b^ Chromosome; ^c^ Position of the QTL; ^d^ The log of odds (LOD) value at the peak likelihood of the QTL; ^e^ Phenotypic variance (%) explained by the QTL; ^f^ Indicates additive, those with positive values show beneficial alleles from parent Linhefenqingdou while those with negative values show beneficial alleles from parent Meng 8206; ^g^ 1-LOD support confidence intervals (confidence interval length); ^h^ Environment where CE represents combined environments and others refer materials and methods; ^i^ References from www.soybase.org.

**Table 3:** Additive and additive × environment interaction effect of QTLs associated with protein and oil contents in soybean seed.

QTL	Chr	Position (cM)	Marker Range	Additive Effect		Additive x Environment Effect						
				A	H^2^ (%)	AE1	AE2	AE3	AE4	AE5	AE6	H^2^ (%)
*qOil-8-4*	8	50.23	bin908-bin909	−0.21 **	7.38	NS	NS	NS	NS	0.29 **	NS	4.11
*qOil-10-2*	10	26.12	bin1134-bin1135	−0.22 **	8.36	NS	NS	NS	−0.12 *	0.22 **	NS	2.40
*qOil-11-1*	11	54.01	bin1274-bin1275	−0.16 **	4.64	NS	NS	−0.10 *	NS	NS	NS	2.18
*qOil-16-1*	16	96.87	bin1819-bin1820	−0.14 **	3.52	NS	NS	NS	NS	NS	0.11 *	0.47
*qPro-6-1*	6	57.91	bin612-bin613	0.38 **	5.55	NS	NS	NS	NS	0.25 *	−0.43 **	2.13
*qPro-7-1*	7	41.68	bin771-bin772	0.59 **	13.47	NS	−0.17 **	NS	−0.12 *	0.55 **	NS	3.17
*qPro-10-1*	10	26.12	bin1134-bin1135	0.34 **	4.62	NS	NS	−0.10 *	NS	0.36 **	NS	1.62

Chr., chromosome. * *p* < 0.05; ** *p* < 0.01; NS, non-significant. A indicates additive effects, those with positive values show beneficial alleles from parent Linhefenqingdou while those with negative values show beneficial alleles from parent Meng 8206.H^2^ indicates phenotypic variation explained by additive effects. AE1, FY2012; AE2, JP2012; AE3, JP2013; AE4, JP2014; AE5, YC2014; AE6, JP2017.

**Table 4:** Estimated epistatic effects (AA) and environmental (AAE) interaction of QTLs for soybean seed oil and protein contents across all environments.

Trait	QTL	Chr_i	Pos_i	Marker Interval_i	QTL	Chr_j	Pos_j	Marker Interval_j	Epistatic (AA) Effect		Epistatic (AA) x Environment Effect						
									AA	H^2^ (%)	AAE1	AAE2	AAE3	AAE4	AAE5	AAE6	H^2^ (%)
Oil	*qOil-2-3*	2	36.37	bin132-bin133	*qOil-13-2*	13	28.91	bin1429-bin1430	−0.14 **	3.81	NS	−0.20 **	NS	NS	NS	0.12 *	0.75
Protein	*qPro-2-1*	2	150.55	bin223-bin224	*qPro-13-3*	13	57.27	bin1455-bin1456	1.65 **	1.06	NS	NS	NS	NS	2.33 **	−2.07 **	0.85
Protein	*qPro-17-2*	17	78.16	bin1892-bin1893	*qPro-17-3*	17	94.43	bin1911-bin1912	0.37 **	0.05	0.32 **	NS	NS	NS	NS	−0.38 **	0.03

Chr_i and Chr_j indicate the two sites involved in epistatic interactions; Pos indicates genetic position for each of the sites. * *p* < 0.05; ** *p* < 0.01; NS, non-significant. AA indicates epistatic effects between two QTLs, those with positive values show two loci genotypes being the same as those in parent Linhefenqingdou (or Meng 8206) have the beneficial effects, while the two-loci recombinants take the negative effects. The case of negative values is the opposite. H^2^ indicates phenotypic variation explained by epistatic effects. AE1, FY2012; AE2, JP2012; AE3, JP2013; AE4, JP2014; AE5, YC2014; AE6, JP2017.

## Supplementary Materials

Supplementary materials can be found at https://www.mdpi.com/1422-0067/20/4/979/s1.

## Abbreviations

RAD-seq	Restriction site associated DNA sequencing
QTL	Quantitative trait loci
RIL	Recombinant inbred line
R^2^	Phenotypic variation explained
GO	Gene ontology
RNA-seq	Ribonucleic acid sequencing
MAS	Marker-assisted selection
Q × E	QTL and environment interaction
RCBD	Randomized complete block design
ANOVA	Analysis of variance
MSG	Multiplexed shotgun genotyping
SNP	Single nucleotide polymorphism
CIM	Composite interval mapping
MCMC	Markov chain monte carlo
