# Supplementary material for: Genome-Wide Detection of Major and Epistatic Effect QTLs for Seed Protein and Oil Content in Soybean Under Multiple Environments Using High-Density Bin Map
Source: Int J Mol Sci. 2019 Feb 23;20(4):979. doi: 10.3390/ijms20040979 (PMC6412760; doi:10.3390/ijms20040979)
Supplement: Supplementary file 1 [file ijms-20-00979-s001.zip › Supplementary Figure S1.docx.pdf]

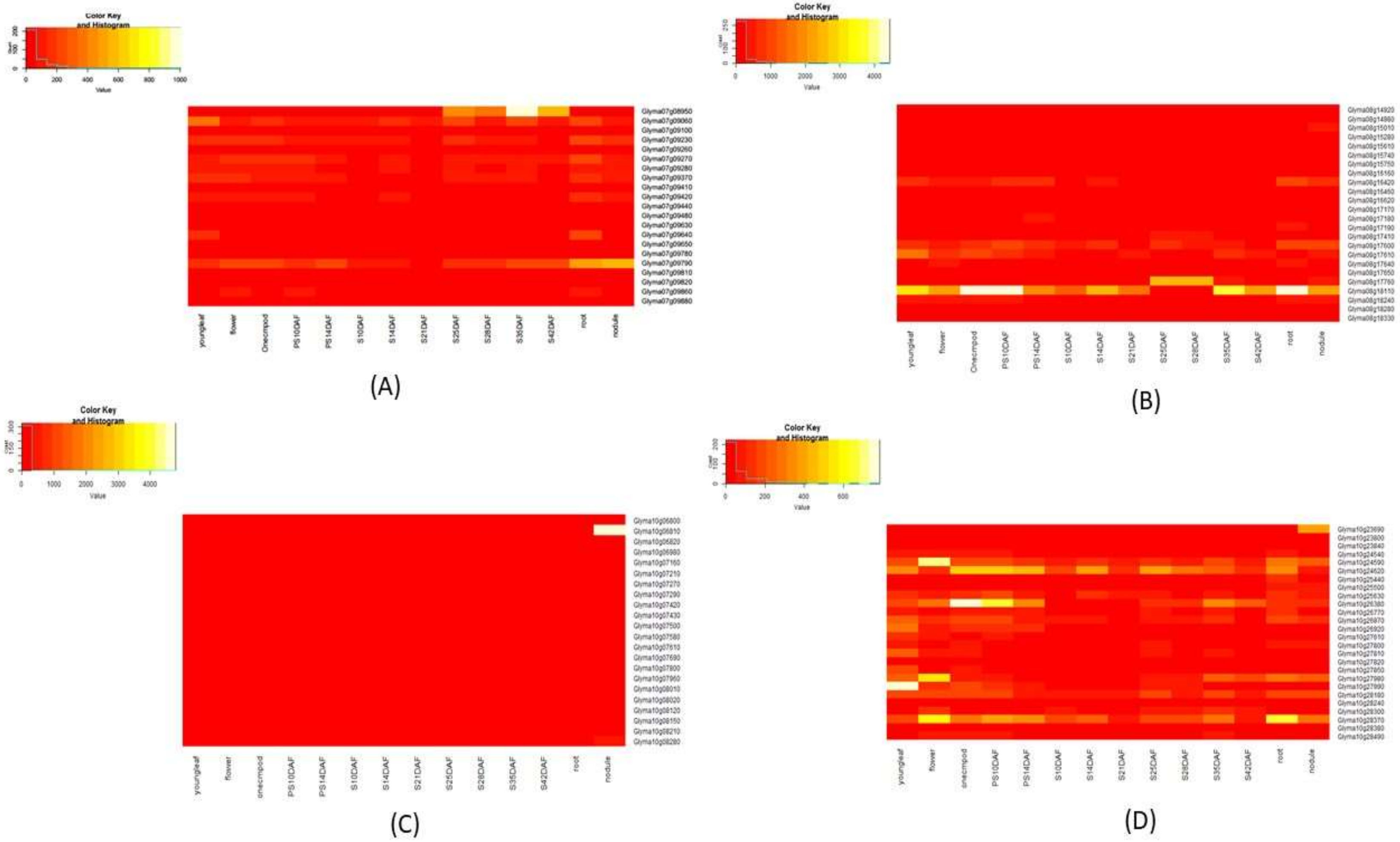

Figure S1. Gene expression analysis of predicated candidate genes among the different soybean tissues and development stages from four major and stable QTLs (**A**) *qPro-7-1*, (**B**) *qOil-8-3*, (**C**) *qOil-10-2* and (**D**) *qOil-10-4*; youngleaf—Young leaf; Onecmpod—1 cm of pod; PS—Pod shell; DAF—Days After flowering; S—Seed.
